# Closed-Loop Control for Fluid Resuscitation: Recent Advances and Future Challenges

**DOI:** 10.3389/fvets.2021.642440

**Published:** 2021-02-23

**Authors:** Behnood Gholami, Wassim M. Haddad, James M. Bailey, William W. Muir

**Affiliations:** ^1^Autonomous Healthcare, Inc., Hoboken, NJ, United States; ^2^School of Aerospace Engineering, Georgia Institute of Technology, Atlanta, GA, United States; ^3^Northeast Georgia Medical Center, Gainesville, GA, United States; ^4^College of Veterinary Medicine, Lincoln Memorial University, Harrogate, TN, United States

**Keywords:** closed-loop control, fluid management, large-volume resuscitation, goal-directed therapy, hemorrhage, hypotension

## Abstract

Fluid therapy is extensively used to treat traumatized patients as well as patients during surgery. The fluid therapy process is complex due to interpatient variability in response to therapy as well as other complicating factors such as comorbidities and general anesthesia. These complexities can result in under- or over-resuscitation. Given the complexity of the fluid management process as well as the increased capabilities in hemodynamic monitoring, closed-loop fluid management can reduce the workload of the overworked clinician while ensuring specific constraints on hemodynamic endpoints are met with higher accuracy. The goal of this paper is to provide an overview of closed-loop control systems for fluid management and highlight several key steps in transitioning such a technology from bench to the bedside.

## Introduction

Fluid therapy is used extensively to treat traumatized patients as well as patients during surgery (1, 2). Fluid therapy is a challenging process involving the consideration of interpatient and intrapatient variability in response to therapy as well as other factors such as comorbidities and general anesthesia. These complexities can result in under- or over-resuscitation (3–5). Given the complexity of the fluid management process, as well as the increased capabilities in hemodynamic monitoring, closed-loop fluid management can reduce the workload of the overworked clinician while ensuring specific constraints on hemodynamic endpoints are met with higher accuracy.

One industry that has witnessed a wide adoption of active (i.e., closed-loop) control technologies is the aerospace industry, where closed-loop control technologies (the *autopilot*) have been used in various aircrafts over the past several decades. More recently, advances in autonomous driving holds the promise of using closed-loop technologies for the driverless operation of vehicles in the near future. Although closed-loop control technologies have been used in various industries for many years, it is not until recently that active control technology has transitioned into medical applications with the majority of applications focusing on pre-clinical or research settings.

## What is A Closed-Loop Control System?

**Figure 1:**
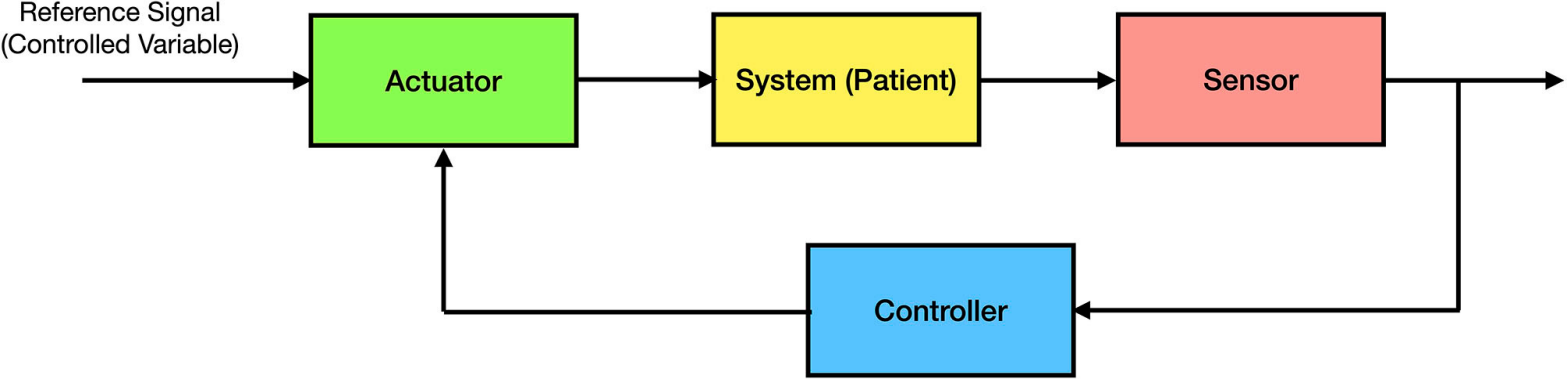
General architecture of a closed-loop control system.

In many systems related problems, a specific variable of interest (the controlled variable) needs to be maintained at a desired value. For example, in the classical inverted pendulum control problem consisting of a pendulum and a moving cart, the goal is to maintain the pendulum in a vertical position by moving the cart horizontally. Every closed-loop control system has three components; namely, (i) a *sensor*, that measures a relevant variable of interest (e.g., the angle of the pendulum relative to the vertical position); (ii) an *actuator* (e.g., electric motor on the cart), that changes the state of the system in order to drive the controlled variable of interest to a desired value; and (iii) a *controller* or *processor*, that uses the sensor measurements in order to compute an appropriate action that drives the actuator to execute the desired control action. The controller can be considered the central processing unit of the system (the “brain”) and, in general, is implemented using proprietary software on a computing device. The general architecture of a closed-loop control system is shown in Figure 1.

Using this architecture, it is straightforward to apply this process to a clinical problem such as fluid management. In this case, the goal would be to maintain the controlled variable of interest at a desired value (e.g., maintaining the cardiac index at 3 L/min/m^2^). In order to design a feedback control system, we need a sensor (e.g., a hemodynamic monitor and its associated sensor to measure cardiac output) and an actuator (e.g., an infusion pump to administer IV fluids) to regulate the trajectory of the controlled variable of interest over time. The control system can be implemented on a computing device such as a computer or a small and limited-resource computing device such as a microcontroller.

An important property of a closed-loop system is system stability. Specifically, closed-loop system stability ensures that system state variables do not significantly deviate from their desired set point values over time. For example, if a developed control system for controlling an inverted pendulum is appropriately designed and the closed-loop system is stable, then we are guaranteed that, for all initial angular positions and velocities relative to the vertical plane, the controlled system will maintain the pendulum at the desired 90-degree angle with respect to the horizontal position of the cart.

Closed-loop stability is an extremely important property for a controlled system. Instability may result in *actuator saturation* (e.g., fluid infusion amplitudes and rates attaining their maximum limits) as well as oscillatory system behavior or, in the worst case, divergent trajectories of the controlled variables. Lyapunov stability theory provides a powerful framework for guaranteeing system stability. Using this framework, an “energy-like” Lyapunov function is constructed and shown that its rate of change with respect to time is negative reflecting the fact that the “energy” of the closed-loop (i.e., controlled) system is decreasing over time (6). An intuitive example of a stable system is a marble placed in a bowl, where, no matter where you release the marble from within the bowl, the marble converges to the point of minimum potential (i.e., energy) corresponding to the bottom point of the bowl (i.e., equilibrium point) after dissipating its kinetic energy.

## Closed-Loop Control in Biomedicine

Although closed-loop systems in biomedicine, including closed-loop drug delivery systems (7), have been designed and investigated as part of research initiatives, a renewed interest in active control has resulted in the development of a number of commercial medical products based on closed-loop control technologies. One notable example is the artificial pancreas, where continuous blood glucose measurements are used to automatically guide the administration of insulin using a computer-controlled insulin pump (8).

The renewed interest in closed-loop technologies for improving performance in clinical applications is due to multiple factors. Until recently, control system designs in biomedicine have been largely based on heuristics (i.e., trial-and-error-based methods). This is due to the complexities inherent in system physiology, wherein mathematical models capturing complex physiological mechanisms of action are not readily developed. Although there has been considerable progress in understanding the pharmacokinetics and pharmacodynamics of drug distribution and drug effect using compartmental models in pharmacology (9), the distribution of fluids using compartmental and volume kinetic models (10–12), and lumped parameter models of blood volume response to fluid infusion (13), such models are approximations to the actual patient physiology. The gap between system modeling and the actual system physiology has been the main limiting factor in the use of rigorous control design frameworks that have been widely used in, for example, the aerospace industry. This is due to the fact that, unlike human or animal physiology, aircraft system dynamics are governed by the well-established laws of aerodynamics and mechanics.

Compartmental models, which are characterized by conservation laws (e.g., conservation of mass, energy, and fluid) involve several simplifying assumptions. One important assumption is that the drug or fluid distribution in the body can be approximated by using only a few *compartments*. A compartment is a macroscopic subsystem involving kinetic homogeneity, where it is assumed that any material entering the compartment is instantaneously mixed with the material in that compartment. Compartmental models have been successfully used to provide approximate models for capturing the behavior of drug and fluid distribution in the body. In addition, such models provide an opportunity for the use of *model-based control design* approaches that use mathematically rigorous closed-loop control techniques to solve specific problems related to drug and fluid distribution management.

Although compartmental models provide the opportunity to leverage model-based control designs, a critical limitation of this design approach is that the patient-specific parameters that define these models are vaguely known. More specifically, compartmental models capture the approximate physiological *structure* of the biomedical system being modeled, whereas the model parameter values (e.g., transfer coefficients between different compartments) are uncertain and vary from patient to patient; these model parameter values are critical in being able to design effective closed-loop, model-based control systems.

Given the aforementioned complexities associated with model-based controller designs for biomedical systems, heuristic approaches have been the dominant control design methods used for controlling these systems. In particular, specific controller design parameters (e.g., the gains of a *proportional-integral-derivative* controller, also known as a PID controller) are “tuned” based on experimental data (14). In this approach, the system physiology is effectively modeled as a “black box,” and the control system parameters are designed (i.e., tuned) by trial and error.

An inherent assumption of heuristic control design methods is that the available data obtained through animal studies (14, 15) or patient population data (16–18) is representative of *all* patients. This approach may be useful when dealing with a problem where a limited understanding of the dynamics or the process to be controlled exists. However, it is predicated on the overly restrictive assumption that all patients are modeled as an “average patient.” Creating an average patient model based on statistical data or limited experimental data has the potential to negatively impact the performance and stability of the closed-loop system when applied to an actual patient.

There have been a number of recent advances that have created an optimal platform for transitioning closed-loop control technologies into a clinical setting. From a theoretical perspective, recent developments in control theory, and specifically, *robust* and *adaptive control*, have allowed for a rigorous control system design of uncertain systems, wherein only an *approximate* system model is used to capture the system physiology and the system parameter values (which are unique to each individual patient) are assumed to lie between upper and lower bounds or are unknown and estimated in real-time. Within this context, compartmental models that capture general fluid or drug distribution can be used to guide the control system design. Examples include using adaptive control technologies to deliver general anesthesia (19, 20) and, more recently, closed-loop control for fluid resuscitation (21).

A second factor that has facilitated the development of advanced closed-loop control technologies is the improved computational power and miniaturization of computer chips. Real-time computation of complex control laws requiring the solution of multiple ordinary differential equations and matrix computations in real-time [e.g., for closed-loop control of fluid resuscitation (21)] on a small computing device was not possible until recently.

Finally, the development of mechanistic models linking the dynamics between biological and physiological laws leading to improved understanding of system physiology (22) and the new paradigm of *in silico* testing and *hardware-in-the-loop simulations* have accelerated the development of closed-loop system design in biomedicine (23). As opposed to testing the design of the control system on animals, which is costly and labor intensive, a mathematical model of the patient can be simulated and the performance of the controller can be tested using *in silico* testing. Specifically, there has been recent interest by the FDA to further leverage the promise of *in silico* models in closed-loop controlled device development (24, 25). The fidelity of *in silico* models have progressed to a level where the FDA recently approved the use of *in silico* testing as a replacement of animal studies prior to testing of an artificial pancreas in humans (26).

## Closed-Loop Control For Fluid Resuscitation

Closed-loop control for fluid resuscitation has been investigated by several research groups. Specifically, closed-loop (i.e., fully automated) and semi-automated fluid management for burn patients has been extensively studied by researchers at the US Army Institute of Surgical Research, San Antonio, TX (27, 28). The semi-automated implementation of this work was later commercialized as part of a clinical decision support tool for fluid management of burn patients. The developed framework, which used urine output rates as the controlled variable of interest, employed a heuristics-based non-linear relationship between the urine output rate and the fluid infusion rate to guide the resuscitation process. A clinical study involving 39 acute burn patients with >20% total body surface area, showed that the developed clinical decision support system reduced the total amount of fluid administered. In addition, a larger number of patients resuscitated by the clinical decision support system met the hourly urine output goals set by the study team.

The authors in (14) investigated the use of their closed-loop control system to guide fluid resuscitation for burn shock in sheep. Specifically, the performance of a PID controller was compared with manual resuscitation when using the urine output rate as the feedback control signal. It was shown that the PID controller was able to produce urine output rates within the desired range with less variation as compared to manual resuscitation. Various controllers based on heuristic design methods such as PID, fuzzy logic, and decision tables have been tested and reviewed elsewhere (29) including studies involving the co-administration of vasopressor and fluids using a closed-loop architecture (30–32).

Another series of recent studies have investigated the use of closed-loop control for goal directed fluid therapy. Specifically, hemodynamic data obtained from patients has been used to develop an algorithm to administer a bolus of fluid (33). The performance of the approach in hemorrhaged pigs was evaluated by comparing the closed-loop system's response to volume depletion with manual fluid resuscitation by anesthesiologists. The study demonstrated that the closed-loop fluid administration system responded appropriately and produced a higher cardiac index as compared to the group which received manual fluid resuscitation by anesthesiologists. The same algorithm was later used as part of a pilot study in humans (18).

While the majority of currently designed closed-loop systems use heuristic approaches to determine fluid administration rates and volumes, a notable exception has been the employment of an adaptive control system predicated on a compartmental model to resuscitate dogs subject to absolute and relative hypovolemia (21). The study involved a total of nine experiments on five dogs of different weights experiencing controlled or uncontrolled hemorrhage as well as cases involving relative and absolute hypovolemia. In this pilot study, it was shown that the adaptive control system could successfully drive stroke volume variation to a predetermined value set by the clinician.

In a recent work, authors in (34) investigate a model-based controller design predicated on a lumped parameter model of blood volume response involving three parameters (13). Specifically, this closed-loop control framework involves a two-step process: a “calibration” phase involving administering an initial fluid bolus and observing the patient's response followed by using a model reference adaptive control architecture to guide fluid infusion. However, as discussed by the authors, the performance of this framework beyond *in silico* tests for 30 randomly generated patients was not investigated. It is also unclear whether the proposed architecture is robust to unmodeled dynamics not captured by the lumped parameter model.

## Discussion

Given the complexity of individual patient physiology under various clinical scenarios and issues associated with under- or over-resuscitation (3–5), designing a closed-loop control system for fluid resuscitation needs to involve a collaborative team effort between control engineers and clinicians. The control system design process needs to employ rigorous control engineering frameworks to address important issues such as system performance, stability, and robustness to modeling uncertainly as well as disturbances (e.g., how the system will respond to rapid changes in patient physiology). Leveraging decades of knowledge gained from physiological modeling, including compartmental models and volume kinetics (10–12), holds a great promise for developing feedback control technologies that will permit a transition from the bench to the bedside.

Clinical insight is critical in designing closed-loop systems for fluid management. The controlled system needs to administer fluids when appropriate, and needs to stop fluid administration when key physiological endpoints are maintained. In the case where the anticipated response to fluid administration is not achieved by the closed-loop system, the system needs to alert the clinician to take over the fluid administration (i.e., control) in much the same way wherein a well-designed autopilot alerts the pilot to take over control of an aircraft.

Prior to transitioning a closed-loop control system for fluid management to a clinical setting, such system needs to be comprehensively tested. This will involve a series of tests ranging from *in silico* testing and hardware-in-the-loop simulation to pre-clinical and clinical testing. In addition, such tests need to assess the system performance in complex clinical scenarios to ensure safety and effectiveness. The clinical and workflow impact of using a closed-loop control system for fluid management in a clinical setting needs to be investigated in future studies.

## Author Contributions

All authors listed have made a substantial, direct and intellectual contribution to the work, and approved it for publication.

## Conflict of Interest

BG has stock ownership in Autonomous Healthcare, Inc. BG was one of the inventors on a patent application (assigned to Autonomous Healthcare Inc.) for a clinical decision support and closed-loop fluid resuscitation system and cardiovascular drug administration. WH has stock ownership in Autonomous Healthcare, Inc. JB has stock options and serves as the Chief Medical Officer in Autonomous Healthcare, Inc. The remaining author declares that the research was conducted in the absence of any commercial or financial relationships that could be construed as a potential conflict of interest.
